# A Rare Case of Suspected Malignant Hyperthermia in a Three-Day-Old Neonate: A Case Report

**DOI:** 10.7759/cureus.102502

**Published:** 2026-01-28

**Authors:** Jad Kabbara, Cham Al Salak, Fawaz Ali, Abdallah Kabbara

**Affiliations:** 1 Anesthesiology, Lake Erie College of Osteopathic Medicine, Erie, USA; 2 Anesthesiology, Damascus University, Damascus, SYR; 3 Anesthesiology, University Hospitals Cleveland Medical Center, Westlake, USA

**Keywords:** dantrolene, hypercarbia, malignant hyperthermia, neonate, volatile anesthetics

## Abstract

Malignant hyperthermia (MH) is a rare, life-threatening pharmacogenetic disorder of skeletal muscle calcium regulation, most commonly triggered by volatile anesthetics and depolarizing muscle relaxants. While pediatric patients are at increased risk compared to adults, MH is exceedingly rare in neonates, and its clinical presentation may differ by age group, with hyperthermia and generalized rigidity more prominent in the youngest patients.

This report describes a three-day-old female who developed a rapid rise in end-tidal CO_2_, tachycardia, metabolic acidosis, and hyperthermia intraoperatively during emergent laparotomy, despite cessation of active warming and stable ventilator settings. These findings, in the context of volatile anesthetic exposure, are consistent with the clinical diagnostic criteria for MH, including unexplained hypercarbia, temperature elevation, and hemodynamic instability. Prompt recognition led to immediate discontinuation of triggering agents, initiation of active cooling, and administration of intravenous dantrolene, resulting in gradual clinical improvement.

This case underscores the importance of early recognition and intervention in suspected MH, even in neonates, highlighting the need for perioperative vigilance, rapid multidisciplinary response, and referral for confirmatory contracture and genetic testing in survivors.

## Introduction

The diagnosis of an acute malignant hyperthermia (MH) reaction is challenging due to the nonspecific and variable nature of its clinical and laboratory features. These manifestations are related to skeletal muscle hypermetabolism and ischemia with systemic sequelae that may include tachycardia, tachypnea, hypercarbia, respiratory and metabolic acidosis, masseter muscle rigidity, generalized muscular rigidity, myoglobinuria, rhabdomyolysis, arrhythmias, cyanosis, skin mottling, hyperkalemia, diaphoresis, rapid temperature elevation, hemodynamic instability, and coagulopathy [1-4]. The clinical presentation can be highly variable, and not all signs are present in every case, which complicates diagnosis. While some studies suggest that hyperthermia and generalized rigidity are prominent in neonates with MH [5], other reports indicate that younger patients may more often present with skin mottling, higher peak lactate levels, lower creatine kinase values, and only a 50% probability of developing generalized rigidity [6]. This highlights the variability in clinical presentation among neonates.

The pathophysiology of MH is most commonly due to inherited variants in the RYR1 gene on chromosome 19, which encodes the skeletal muscle ryanodine receptor, a calcium release channel of the sarcoplasmic reticulum [2]. However, in some families, MH susceptibility is not linked to this locus, indicating genetic heterogeneity [4]. The GeneReviews expert group notes that the majority of cases are associated with RYR1 mutations, but other loci may be involved [1,7].

Regarding anesthetic management, nontriggering anesthetics (such as intravenous agents and regional anesthesia) are considered safe for individuals with MH susceptibility [1]. The Malignant Hyperthermia Association of the United States (MHAUS) and the GeneReviews expert group both state that routine prophylactic dantrolene pretreatment is no longer recommended for MH-susceptible patients undergoing nontriggering anesthesia [4]. This reflects current consensus in the medical literature.

The incidence of MH ranges from one in 4,500 with the use of succinylcholine to one in 60,000 anesthetics without succinylcholine. The risk is highest in children and young adults, with the incidence decreasing after age 50 and being rare in infants and neonates. This age-related distribution is supported by large epidemiologic studies and registry data, which also note that the occurrence of acute MH in neonates is rare, a finding paralleled in the swine model. Variability in gene penetrance, particularly among RYR1 mutation carriers, affects individual susceptibility and clinical expression of MH, with incomplete and sex-dependent penetrance observed in human studies. These points are supported by the medical literature, including the GeneReviews expert group and recent multicenter genetic studies [1,7-9].

We are reporting a suspected case of MH in a three-day-old girl.

## Case presentation

A three-day-old female was born by spontaneous vaginal delivery at 36 weeks of gestation to a gravida 3, para 1 (G3P1011) mother with a history of insulin-dependent diabetes mellitus, complicated by pregnancy-induced hypertension and rupture of membranes six hours prior to delivery. Birth weight was 2,946 g, and appearance, pulse, grimace, activity, and respiration (Apgar) scores were seven and nine at one and five minutes, respectively. On the third day of life, the baby, who had not yet passed meconium, developed abdominal distention and signs of peritonitis and grew gram-negative bacteremia; the specific organism was not identified in the available medical records. She was electively intubated by the neonatal intensive care unit (NICU) staff due to increasing work of breathing and clinical deterioration. She was intubated with a 3.5 tube taped at 9 cm at the lips and ventilated on synchronized intermittent mandatory ventilation (SIMV) mode with 30% FiO_2_ at a rate of 16 and pressure of 18/4.

Perioperative laboratory evaluation demonstrated elevated hematocrit and hemoglobin with otherwise normal electrolytes and renal function (Table 1). Perioperative vital signs were blood pressure (BP) 63/37 mmHg, heart rate 154 bpm, respiratory rate 16 breaths/min, and temperature 36.8°C. The baby had an arterial line, a 24-g peripheral line, a Foley catheter, a rectal tube, and a nasogastric tube. She was rushed for an exploratory laparotomy after obtaining maternal consent; no family history of anesthesia complications was reported. It is important to note that prior to anesthesia, the patient was already mildly acidotic and tachycardic due to gram-negative sepsis, which complicated intraoperative assessment and the differential diagnosis for MH.

**Table 1:** Perioperative laboratory values prior to surgery with corresponding neonatal reference ranges.

Parameter	Value	Reference range (neonate)
Hematocrit (%)	56.7	45-65
Hemoglobin (g/dL)	19.5	14-24
Platelets (×10³/µL)	212	150-400
White blood cells (×10³/µL)	12	9-30
Sodium (mEq/L)	137	135-145
Potassium (mEq/L)	Hemolyzed	3.5-6.0
Chloride (mEq/L)	100	98-108
Bicarbonate (mEq/L)	18	20-28
Blood urea nitrogen (mg/dL)	8	5-15
Creatinine (mg/dL)	0.9	0.3-1.0
Glucose (mg/dL)	116	45-125

In the OR, endotracheal tube placement was confirmed by equal bilateral lung auscultation and positive end-tidal CO_2_ (EtCO_2_) via capnography. The baby was administered 0.4 mg of pancuronium and 20 mcg of fentanyl. Standard monitoring was applied, and invasive arterial BP was maintained. She was ventilated with pressures of 22/4 at a rate of 20; flow consisted of 1 L O_2_ and 1 L air with 0.5% isoflurane. A pediatric circuit was used, and fluids included normal saline with NICU electrolytes at a maintenance rate of 18 cc/h. Initial operating room (OR) vitals: BP 50s/30s mmHg, HR 155-160 bpm, temperature 37.1°C (esophageal probe), FiO_2_ 53%, SpO_2_ 99-100%, and EtCO_2_ 32 mmHg. No additional antibiotics were administered, as none were due at that time.

Surgical findings included a perforated jejunum requiring jejunal resection with jejunostomy, appendectomy, and a rectal biopsy to rule out Hirschsprung disease. At 45 minutes into the procedure, the BP began to drop with associated tachycardia, prompting transfusion of 20 cc of type-matched, irradiated packed red blood cells. A blood gas analysis 45 minutes after transfusion showed pH 7.34, PCO_2_ 25 mmHg, PO_2_ 206 mmHg, HCO_3_ 13.4 mEq/L, base excess -10.2, and hematocrit 46%; hemodynamically, the baby was stable (Table 1).

At 3.5 hours into surgery near skin closure, the temperature rose from 37.1°C to 37.6°C, and EtCO_2_ increased despite no changes to ventilator settings or warming. The first concerning sign was EtCO_2_ rising despite increasing the ventilator rate to 24, followed by a rapid temperature increase. An arterial blood gas showed severe acidemia and marked hypercapnia (Table 2). The temperature peaked at 38.7°C; due to this rise in temperature as well as the hypercapnia, MH was suspected, prompting discontinuation of isoflurane, flushing of the circuit, and administration of 100% O_2_. Bilateral breath sounds were confirmed, indicating proper tube placement and adequate perfusion to both lungs, and the baby was exposed and cooled with ice water and towels. Dantrolene was administered at 2.5 mg/kg and repeated at the same dose.

**Table 2:** Each column reflects the patient’s physiologic parameters at specific intervals during the acute MH episode, allowing direct comparison with narrative descriptions in the text. Missing values were not obtained or not recorded at those time points. MetroHealth Medical Center. Intraoperative laboratory records. Cleveland, OH; circa 2008. MH, malignant hyperthermia

Parameter	18:33	20:15	20:45	21:15	21:31	Reference ranges
pH (7.35-7.45)	7.34	6.82	6.67	6.88	7.06	7.35-7.45
PCO₂ (35-45 mmHg)	25	122.6	198.7	118	65	35-45
PO₂ (50-80 mmHg)	206	131.6	96.5	240	322	50-80
HCO₃ (18-24 mEq/L)	13.4	21.2	22.4	22.5	18	18-24
Base excess (-2 to +2 mmol/L)	-10.2	-14.9	-17.8	-13.5	-12.5	-2 to +2
Hematocrit (45-65 %)	46	42	39	38	38	45-65
Calcium (1.10-1.30 mmol/L)	-	1.3	1.24	1.3	1.23	1.10-1.30
Potassium (3.5-6.0 mEq/L)	-	3.6	4.7	3.3	3.4	3.5-6.0
Glucose (45-125 mg/dL)	-	213	223	284	273	45-125
FiO₂ (21-100%)	53	100	100	100	100	21-100

Chest rise became asymmetrical, and auscultation revealed decreased breath sounds on the left. A chest X-ray showed collapse of the left lung and the right upper lobe. Endobronchial intubation was suspected, and the tube was repositioned, restoring equal bilateral breath sounds. Repeat X-ray showed good bilateral lung expansion, and the temperature gradually decreased to 36°C, but EtCO_2_ remained high. Hand-bag ventilation with 100% O_2_ via a fresh circuit was initiated, and blood gases initially worsened but then improved. The NICU attending was called along with the MH hotline, and the baby was transitioned to oscillatory ventilation on 100% O_2_ with amplitude 67 and mean arterial pressure 19.

A total dose of 10 mg/kg of dantrolene was given in divided doses, and fluid boluses were administered in increments of 10 cc/kg. Bicarbonate was given at 1 mEq/kg and mannitol at 0.5 g/kg. Kidney, blood count, metabolic panel, coagulation studies, creatine kinase (CK), lactate, and urine myoglobin were all ordered. Total fluid administered was 230 cc, total blood products 20 cc, estimated blood loss 6 cc, and urine output 15 cc. At 10:30 PM, after 90 minutes on the oscillator, the baby was transferred to the NICU with monitors and the same oscillator settings. She remained intubated and afebrile, and her carbon dioxide levels remained in the 50s. Eight hours after surgery, carbon dioxide levels normalized to 36, lactate was 0.6 mg/dL, CK was 200 IU/L, and no myoglobin was detected in the urine. Her ventilatory needs gradually decreased, and she was successfully extubated on the fourth postoperative day.

On postoperative day 15, the baby was taken to the OR for Broviac placement and a muscle biopsy under total intravenous anesthesia using propofol, ketamine, and rocuronium. She remained stable, and the procedure was uneventful. Due to limited resources at our facility, the muscle specimen was only examined microscopically. A formal caffeine-halothane contracture test (CHCT) was not performed. The pathology report showed no evidence of myopathy or other identifiable muscle abnormalities. Histology revealed no specific changes such as those seen in central core disease or other neuromuscular disorders. After the muscle biopsy was obtained, an attempt to acquire genetic testing from the family was made multiple times; however, the family refused.

## Discussion

The management of MH-susceptible patients requires strict avoidance of all known triggers, specifically all volatile inhalational anesthetics (except nitrous oxide) and succinylcholine, as emphasized by the American Society of Anesthesiologists and the Society for Ambulatory Anesthesia. Preoperative preparation of the anesthesia machine should include removal of vaporizers, replacement of all disposable components (breathing circuit, reservoir bag, and CO_2_ absorbent), and flushing the system with high fresh gas flows (e.g., 10 L/min of oxygen). The use of activated charcoal filters can further reduce trace volatile anesthetic concentrations and is recommended for rapid preparation [3,10].

In the case of this specific patient’s management, multiple differential diagnoses were considered as the case unfolded, with alternative explanations for the intraoperative findings including septic shock, preexisting metabolic derangements, and unilateral ventilation due to selective endotracheal tube placement. Due to the patient’s prior infection that grew gram-negative bacteremia, septic shock was included in the differential diagnosis, as was endobronchial intubation, which could contribute to tachycardia and possible hypercarbia. Although there was a plethora of possible differential diagnoses, the anesthesiologist and the team targeted the diagnosis of MH based on the clinical evidence present at the time. Due to the symptoms occurring after administration of anesthesia, which presented as tachycardia, hypercarbia, and fever, MH was suspected because of the rapid rise in EtCO_2_ and temperature in the context of volatile anesthetic exposure, along with resolution following dantrolene, all of which supported a presumptive diagnosis of MH. A rapid infusion with an aggressive dose of 2.5 mg/kg dantrolene was administered to prevent further symptoms of MH, such as muscle rigidity [11-14]. The lack of muscle rigidity in our patient’s case was suspected to be due to the rapid administration of dantrolene and the immediate withdrawal of the offending agent. Furthermore, our patient demonstrated only mild temperature elevation and no documented muscle rigidity, which aligns with literature noting variable expression in neonates and underscores the importance of recognizing alternative early signs, such as hypercarbia and metabolic acidosis [2]. The suspicion of MH was further supported as the patient’s symptoms, including hypercarbia and mild hyperthermia, improved following dantrolene administration and active cooling. Heart rate remained elevated during the acute intraoperative phase, peaking at 160 bpm, and subsequently normalized with dantrolene and correction of metabolic disturbances, consistent with the expected physiological response in MH.

Dantrolene is the only specific treatment for MH. The American Society of Anesthesiologists and the Society for Ambulatory Anesthesia recommend immediate administration of dantrolene at the first clinical suspicion of MH, as delays are associated with increased morbidity and mortality [7]. The initial recommended dose is 2.5 mg/kg IV, repeated as needed until symptoms resolve, with a typical upper limit of 10 mg/kg, though higher doses may be used if necessary [9]. If there is no response after 10 mg/kg, alternative diagnoses should be considered. Overcooling should be avoided, as dantrolene often leads to rapid temperature reduction; external cooling should be titrated to avoid hypothermia [10].

Regarding pediatric susceptibility, MH can occur at any age, including infancy, and a previous uneventful anesthetic does not exclude risk [8]. Family history is variable, and a significant proportion of both pediatric and adult patients have had prior uneventful anesthetics [1].

Measurement of CK is not a reliable diagnostic tool for MH susceptibility. While CK may be elevated during or after an acute MH episode due to rhabdomyolysis, it can also be normal in MH-susceptible individuals, especially if dantrolene is administered promptly [5]. Conversely, CK can be elevated for other reasons, such as surgical muscle injury or non-MH myopathies, limiting its specificity and sensitivity for MH diagnosis [12]. The relatively low CK levels observed in this patient's case may reflect early intervention with dantrolene, the use of nondepolarizing muscle relaxants, or limited muscle mass in a neonate, rather than the absence of MH susceptibility. Similar neonatal MH cases have been reported, highlighting variable presentations including early hypercarbia and metabolic acidosis without overt rigidity, emphasizing the importance of early recognition and intervention [13,15].

Genetic testing for pathogenic variants in RYR1 and CACNA1S can identify MH susceptibility in some families, but current genetic testing does not detect all susceptible individuals and cannot yet replace the CHCT as the definitive diagnostic test [13].

The CHCT remains the gold standard for diagnosis of MH susceptibility, with high sensitivity and acceptable specificity [4]. However, a small percentage of clinically suspected patients may test negative, in which an alternative diagnostic method, the in vitro contracture test (IVCT), may also be employed where available [11,13].

In summary, management of MH-susceptible patients centers on strict trigger avoidance, meticulous anesthesia machine preparation, and immediate dantrolene administration upon suspicion of MH, as outlined by the American Society of Anesthesiologists and the Society for Ambulatory Anesthesia [3,10].

## Conclusions

This case underscores the diagnostic and management challenges of MH in neonates, a rare but potentially fatal condition if unrecognized or untreated. The clinical presentation in this 3-day-old infant, characterized by rapid onset of hypercarbia, tachycardia, metabolic acidosis, and hyperthermia during exposure to volatile anesthetics, mirrors the age-specific features described in recent pediatric MH cohorts, where temperature elevation and generalized rigidity are prominent in the youngest patients. 

Prompt recognition and immediate intervention with discontinuation of triggering agents, supportive measures, and dantrolene administration remain the cornerstone of management and are associated with improved outcomes. This case further highlights the importance of perioperative vigilance, multidisciplinary preparedness, and the need for confirmatory testing and genetic counseling in survivors and their families. In summary, this report adds to the growing body of evidence that MH can occur in neonates, and early diagnosis and evidence-based management are essential to optimize outcomes in this vulnerable population.

## References

[1] Otsuki S, Miyoshi H, Mukaida K, Yasuda T, Nakamura R, Tsutsumi YM (2022). Age-specific clinical features of pediatric malignant hyperthermia: a review of 187 cases over 60 years in Japan. Anesth Analg.

[2] Kilmartin E, Grunwald Z, Kaplan FS, Nussbaum BL (2014). General anesthesia for dental procedures in patients with fibrodysplasia ossificans progressiva: a review of 42 cases in 30 patients. Anesth Analg.

[3] Frassanito L, Sbaraglia F, Piersanti A (2023). Real evidence and misconceptions about malignant hyperthermia in children: a narrative review. J Clin Med.

[4] Litman RS, Rosenberg H (2005). Malignant hyperthermia: update on susceptibility testing. J Am Med Assoc.

[5] Rosenberg H, Pollock N, Schiemann A, Bulger T, Stowell K (2015). Malignant hyperthermia: a review. Orphanet J Rare Dis.

[6] Pinyavat T, Riazi S, Deng J, Slessarev M, Cuthbertson BH, Ibarra Moreno CA, Jerath A (2024). Malignant hyperthermia. Crit Care Med.

[7] Riazi S, Biesecker L, Rosenberg H (1993). Malignant Hyperthermia Susceptibility. https://www.ncbi.nlm.nih.gov/books/NBK1146/.

[8] Ibarra Moreno CA, Hu S, Kraeva N (2019). An assessment of penetrance and clinical expression of malignant hyperthermia in individuals carrying diagnostic ryanodine receptor 1 gene mutations. Anesthesiology.

[9] Larach MG, Klumpner TT, Brandom BW (2019). Succinylcholine use and dantrolene availability for malignant hyperthermia treatment: database analyses and systematic review. Anesthesiology.

[10] Urman RD, Rajan N, Belani K, Gayer S, Joshi GP (2019). Malignant hyperthermia-susceptible adult patient and ambulatory surgery center: Society for Ambulatory Anesthesia and Ambulatory Surgical Care Committee of the American Society of Anesthesiologists position statement. Anesth Analg.

[11] Allen GC, Larach MG, Kunselman AR (1998). The sensitivity and specificity of the caffeine-halothane contracture test: a report from the North American Malignant Hyperthermia Registry. Anesthesiology.

[12] Sudo RT, Cunha LB, Carmo PL (2010). Use of the caffeine-halothane contracture test for the diagnosis of malignant hyperthermia in Brazil. Braz J Med Biol Res.

[13] Stowell KM (2014). DNA testing for malignant hyperthermia: the reality and the dream. Anesth Analg.

[14] Riazi S, Kraeva N, Hopkins PM (2018). Updated guide for the management of malignant hyperthermia. Can J Anaesth.

[15] Nnamani NP, Moss DR (2015). Babies in distress: malignant hyperthermia in infancy explored. Clin Pediatr (Phila).

